# Physical Vapor Deposition of High-Mobility P-Type Tellurium and Its Applications for Gate-Tunable van der Waals PN Photodiodes

**DOI:** 10.1021/acsami.4c14865

**Published:** 2024-12-20

**Authors:** Tianyi Huang, Sen Lin, Jingyi Zou, Zexiao Wang, Yibai Zhong, Jingwei Li, Ruixuan Wang, Zhixing Wang, Kevin St. Luce, Rex Kim, Jianzhou Cui, Han Wang, Qing Li, Min Xu, Sheng Shen, Xu Zhang

**Affiliations:** 1 Department of Mechanical Engineering, Carnegie Mellon University, Pittsburgh, Pennsylvania 15213, United States; 2 Department of Electrical and Computer Engineering, Carnegie Mellon University, Pittsburgh, Pennsylvania 15213, United States; 3 Ming Hsieh Department of Electrical and Computer Engineering, University of Southern California, Los Angeles, California 90089, United States; 4 Ray and Stephanie Lane Computational Biology Department, School of Computer Science, Carnegie Mellon University, Pittsburgh, Pennsylvania 15213, United States; 5 Department of Materials Science and Engineering, Carnegie Mellon University, Pittsburgh, Pennsylvania 15213, United States

**Keywords:** gate-tunable, van der Waals materials, heterojunction, PN diodes, high mobility, photodetectors, tellurium

## Abstract

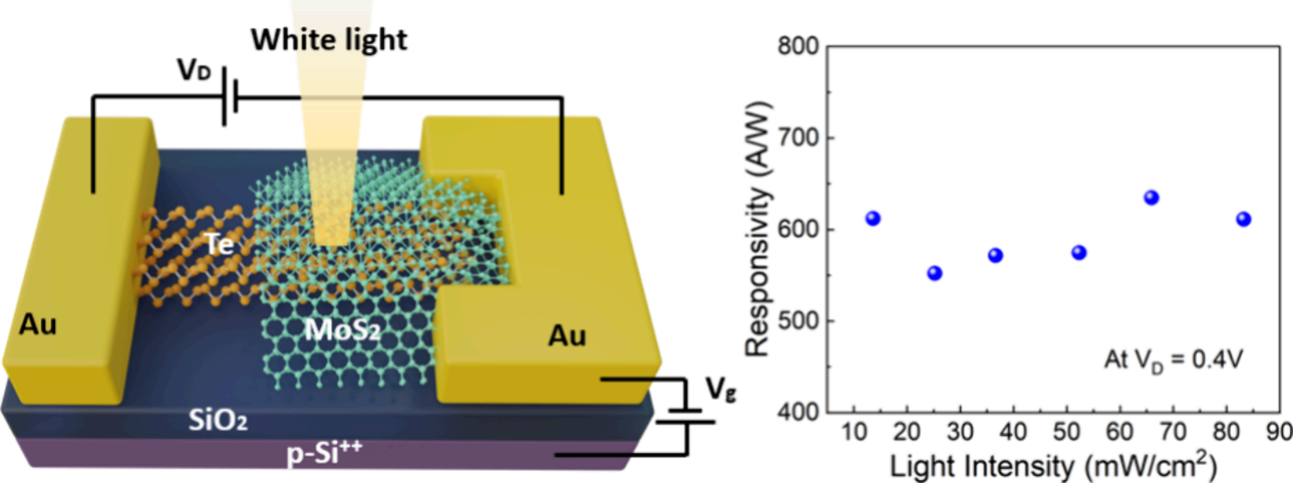

Recently, tellurium has attracted resurgent interest due to its outstanding p-type characteristics and ambient environmental stability. Here, we present a substrate engineering-based physical vapor deposition method to synthesize high-quality Te nanoflakes and achieved a field-effect hole mobility of 1450 cm^2^/(V s), which is, to the best of our knowledge, the highest among existing synthesized two-dimensional p-type semiconductors. The high mobility of Te enables the fabrication of Te/MoS_2_ PN diodes with highly gate-tunable characteristics. The Te/MoS_2_ heterostructure is demonstrated to be used as visible-light photodetectors with a current responsivity up to 630 A/W, which is about 1 order of magnitude higher than one achieved using p-type Si-MoS_2_ PN photodiodes. The photoresponse of Te/MoS_2_ heterojunctions also exhibits strong gate tunability due to their ultrathin thickness and unique band alignment. The successful synthesis of high-mobility Te and its integration into Te/MoS_2_ photodiodes show promise for the development of highly tunable and multifunctional photodetectors.

## Introduction

PN diodes, typically made of a junction between p-type and n-type semiconductors, are one of the most fundamental building blocks of electronics,^1^ with a wealth of applications in photovoltaics,^2^ integrated circuits,^3,4^ and photodetectors.^5^ Since the first successful isolation of graphene,^6^ two-dimensional (2D) van der Waals semiconductors^7^ have emerged as promising candidates that could potentially transform traditional silicon-based electronics with new form factors, such as flexible, ultralight, and/or transparent circuits in the post-Moore era.^8,9^ Despite substantial advances in the synthesis and engineering of n-type 2D semiconductors, superior and stable p-type 2D semiconductors have been much less explored.^10^ Metal oxides,^11^ as an alternative family of emerging semiconductors, are also short of high-quality p-type members. It hindered the development of a diverse portfolio of 2D PN diodes in which p-type semiconductors are essential components. Tellurium (Te) is an elemental p-type van der Waals semiconductor, possessing 1D helical chains of covalent bonds and the adjacent chains packed via van der Waals interactions in a hexagonal lattice.^12−16^ Studies on Te attract considerable resurgent interest especially due to its excellent environmental stability,^12,14^ which is a clear advantage compared with black phosphorus (BP), which is one of the most extensively studied 2D p-type semiconductors.^17−19^

Due to its unique chiral-chain structure and relatively strong bonding, it is extremely difficult, if not impossible, to mechanically exfoliate Te flakes from its bulk form like other van der Waals materials.^20^ Wang et al. used a hydrothermal method to synthesize Te nanoflakes through the reduction of sodium tellurite (Na_2_TeO_3_) and achieved a hole mobility of 700 cm^2^ V^–1^ s^–1^.^12^ However, the involvement of organic solvents often introduces impurities and surface trap states during the synthesis and suffers from limited scalability. As an alternative technique, hydrogen-assisted chemical vapor deposition (CVD) has also been demonstrated to synthesize 2D Te flakes on mica substrates.^21^ Zhao et al. also showed a chemical vapor transport method to synthesize 2D Te flakes with a thickness down to 70 nm and with a mobility of 379 cm^2^ V^–1^ s^–1^.^22^ Yang et al. significantly improved the hole mobility of synthesized Te nanobelts up to 1370 cm^2^ V^–1^ s^–1^ using a CVD method through high-temperature reduction of TiO_2_.^23^ Meanwhile, Zhou et al. showed that Te nanostructures can be obtained by molecular beam epitaxy (MBE) at low temperature (≤120 °C) and exhibited field-effect mobilities up to 707 cm^2^ V^–1^ s^–1^.^24^ Recently, physical vapor deposition (PVD) emerged as an alternative promising strategy to synthesize Te in a scalable manner.^25−29^ Compared with CVD approaches, the PVD method typically allows relatively low synthesis temperatures and therefore a wider range of compatible substrates. In addition, PVD methods also require a shorter deposition time and a facile gas flow control setup as well as produce minimal hazardous byproducts.^30^ For example, Zhao et al. demonstrated that thermal evaporation at cryogenic temperatures can produce wafer-scale Te thin films with an effective hole mobility of ∼35 cm^2^ V^–1^ s^–1^.^29^ Controllable unbalanced magnetron sputtering can also be used to obtain ultrathin Te films with a carrier mobility of up to 19 cm^2^ V^–1^ s^–1^.^25^ Despite these recent progresses, the mobility of as-synthesized Te still has plenty of room to improve.

In this work, we developed a PVD method with substrate engineering to synthesize high-quality Te nanoflakes and achieved a field-effect hole mobility of 1450 cm^2^/(V s), which is, to the best of our knowledge, the highest value among existing synthesized Te and other alternative 2D p-type semiconductors. Compared with the traditional CVD process, our PVD method reduces the deposition temperature by nearly 90–140 °C and the deposition time by more than 50%.^23^ Substrate engineering is a widely used strategy in CVD approaches to facilitate the oriented growth of 2D materials. Here, by introducing atomically flat hexagonal boron nitride (hBN) as growth substrates in PVD systems, we found that p-type Te with an ultrahigh hole mobility can be synthesized. To demonstrate the high quality of as-synthesized Te, we built a Te-MoS_2_ 2D heterojunction as a PN diode, which shows strong gate tunability. The rectification ratio of the Te-MoS_2_ diode can reach 4000 and can be electrostatically tuned through gating. Under white-light illumination, the Te-MoS_2_ can be used as a photodetector with a current responsivity up to 630 A/W, which is about 1 order of magnitude higher than the one achieved using p-type Si-MoS_2_ PN photodiodes.^31^ Importantly, under different gate bias, the responsivity of the Te-MoS_2_ diode exhibits a different dependence on light power density. The tunable photoresponse of the Te-MoS_2_ diode can potentially open up new opportunities in building ultralight and multifunctional optoelectronic devices.

## Results and Discussion

**Figure 1:**
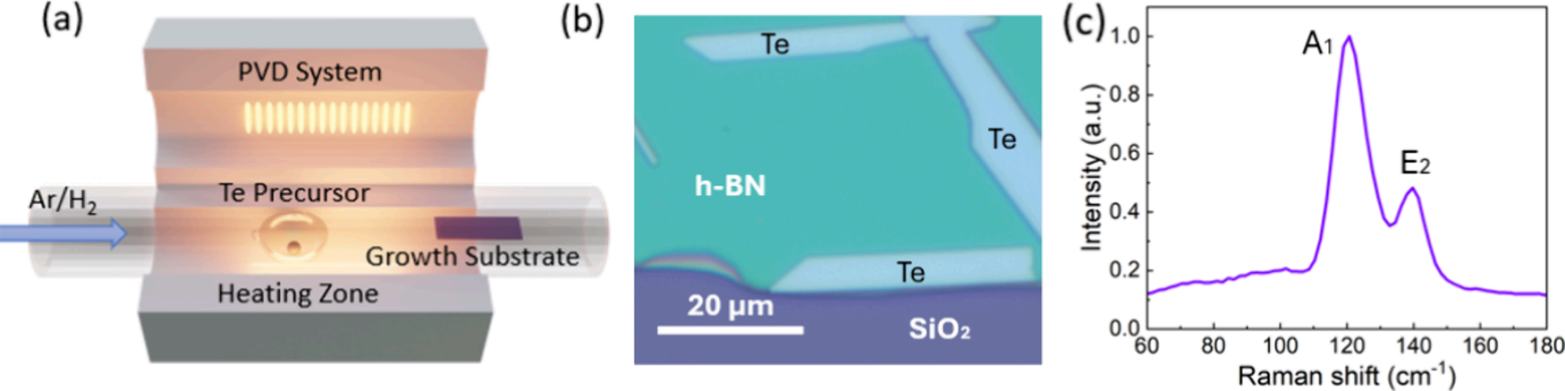
(a) Schematic of the PVD synthesis setup for 2D Te nanoflake growth. (b) Optical image of the as-synthesized Te flakes. (c) Raman spectrum of the PVD-grown Te flakes.

Here, ultrathin Te was synthesized by the PVD approach, as illustrated in Figure 1a. The hBN flakes were mechanically exfoliated onto a piece of a silicon wafer capped with 285 nm-thick SiO_2_, serving as the atomically smooth growth substrate. Bulk Te (from Sigma-Aldrich) was used as the precursor placed in a quartz boat, and the growth substrate was loaded in the downstream region about 9 in. away from the Te precursor (Figure 1a). A mixture of argon and hydrogen was used as the carrier gas to deliver the precursor vapor for synthesis. Detailed experimental parameters can be found in the Experimental Methods. Figure 1b shows a typical optical image of as-synthesized Te flakes on the hBN substrates. Raman characterization shows two peaks at 120 cm^–1^ of the A_1_ mode and 140 cm^–1^ of the E_2_ mode (Figure 1c), which are consistent with the literature.^23,32^

**Figure 2:**
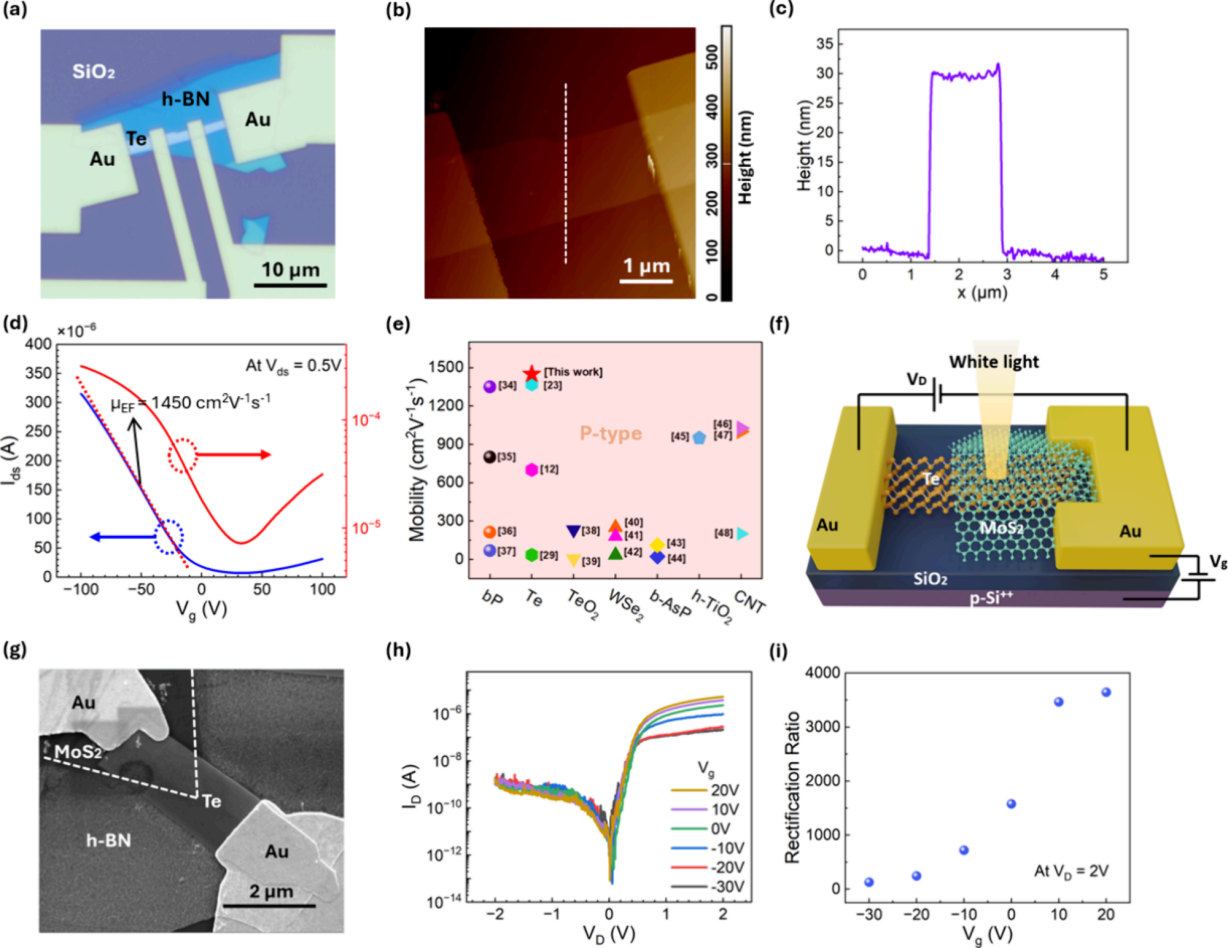
(a) Optical image of the as-fabricated four-point Te FET. (b) AFM scanning image of the Te FET. (c) AFM height profile of the Te FET channel (along a white dashed line). (d) *I*–*V* transfer curve of the Te FET (using the two electrodes in the middle as source and drain contacts, *V*_ds_ = 0.5 V) in both linear (blue line) and logarithmic (red line) scales. (e) Measured field-effect mobility of the Te FET, benchmarked with reported p-type 2D semiconductors and CNT. (f) Schematic of the Te-MoS_2_ van der Waals PN heterojunction-based photodiode. (g) SEM image of one representative Te-MoS_2_ heterojunction diode. The shape of the MoS_2_ flake is indicated by the white dashed line. (h) Measured channel current *I*_D_ (on a logarithmic scale) as a function of channel bias *V*_D_ at various gate voltages. (i) Calculated rectification ratio as a function of gate voltage *V*_g_ at *V*_D_ = 2 V.

Field-effect transistors (FETs) with four-point contacts were then fabricated based on the PVD-synthesized Te to characterize its transport properties (Figure 2a). Gold (Au) was used as the source/drain electrodes, and a heavily doped silicon substrate was used as the back-gate terminal. The thickness of the Te channel was determined by atomic force microscopy (AFM, Figure 2b,c). The Te channel has a smooth surface and a uniform thickness of about 30 nm. Quasi-static *I*–*V* characterization of the Te FET was carried out using a semiconductor parameter analyzer (B1500A). The *I*–*V* transfer curves in both linear and logarithmic scales are shown in Figure 2d, under a drain bias *V*_ds_ of 0.5 V and a back-gate bias swept from −100 to 100 V. The *I*–*V* measurement confirms the p-type behavior of Te for *V*_g_ < 30 V. The transfer curve of the Te FET exhibits ambipolar characteristics, indicating a relatively small band gap of Te, which is also consistent with the literature.^12,23,33^ The on/off ratio of this FET is around 44. The field-effect hole mobility can be extracted from eq 1.
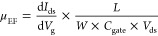
1where *I*_ds_ is the channel current, *V*_g_ is the back-gate bias, *L* is the channel length, *W* is the channel width, *C*_gate_ is the capacitance between the channel and the back-gate per unit area, and *V*_ds_ is the drain bias. The hole mobility of the Te FET was estimated to be 1450 cm^2^ V^–1^ s^–1^. We benchmarked our result with state-of-the-art p-type semiconductors in Figure 2e.^12,23,29,34−48^ From a materials perspective, the development of large-area, high-quality, continuous Te films with precise thickness uniformity is crucial for future high-density, large-array applications. To achieve this, future work on refining synthesis parameters, particularly growth time and temperature, to enhance the coverage and uniformity of the resulting Te samples will be needed. Furthermore, tellurium’s relatively low melting point (449.5 °C)^49^ offers an opportunity to further lower the growth temperature, which holds potential for developing CMOS-compatible PVD synthesis methods for Te films. This would be a key step toward direct Te growth on prepatterned structures and, ultimately, the heterogeneous integration of Te-based electronic devices with other functional modules, all while maintaining CMOS-compatible processing temperatures without affecting front-end-of-line (FEOL) processes.

**Figure 3:**
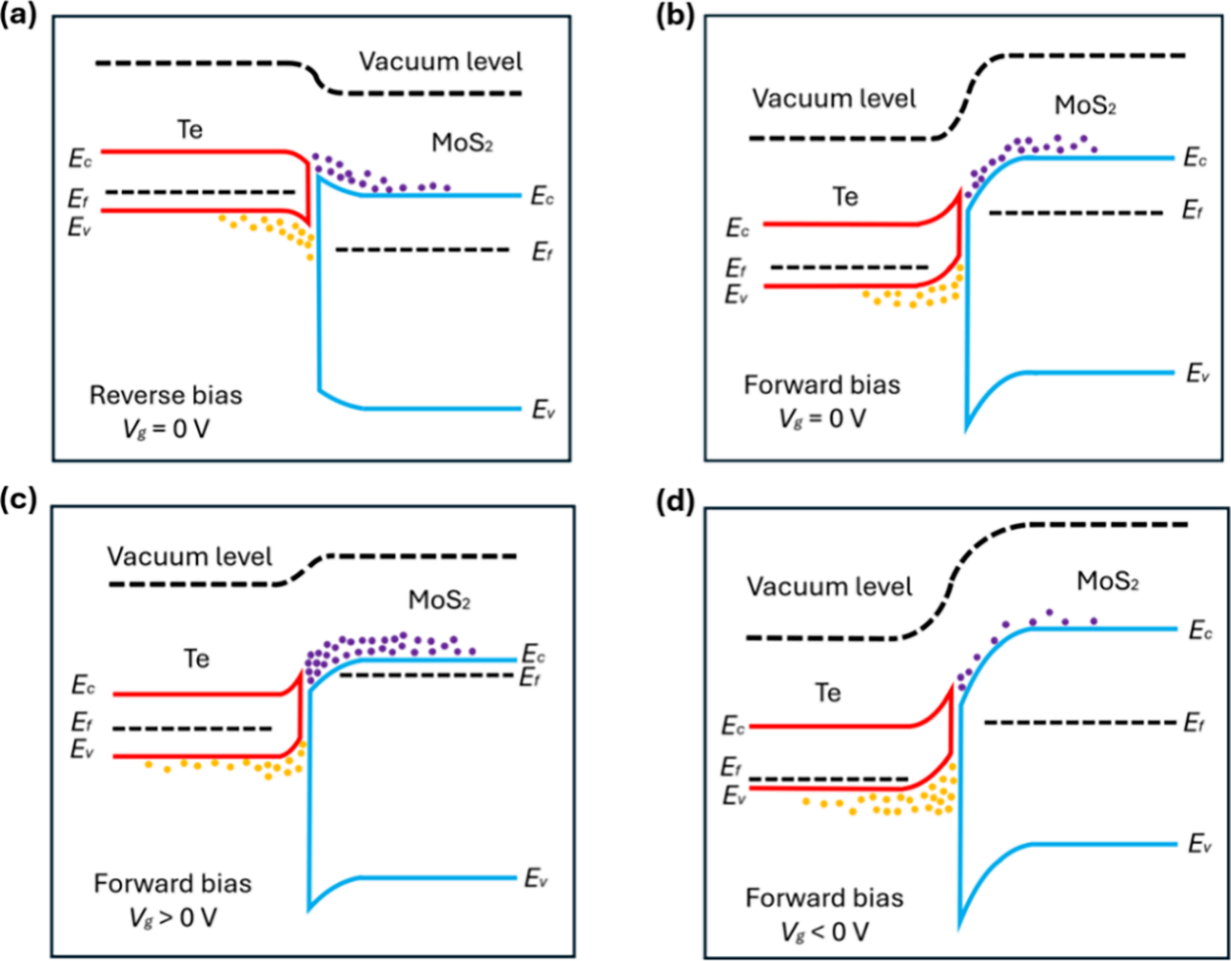
Band structure of the Te-MoS_2_ van der Waals PN heterojunction under reverse (a) and forward (b) voltage biases without applying a gate voltage. The majority of carriers in MoS_2_, i.e., electrons, are indicated as purple-colored dots. The majority of carriers in Te, i.e., holes, are indicated as yellow-colored dots. Electrons in MoS_2_ show different energy barriers at the MoS_2_/Te interface under forward and reverse biases, resulting in a current rectification phenomenon. (c) Band structure of the heterojunction under a forward voltage bias with a positive gate voltage applied. (d) Band structure of the heterojunction under a forward voltage bias with a negative gate voltage applied.

The synthesis of high-mobility p-type Te flakes also makes it possible to build van der Waals PN heterojunctions as photodiodes. The high mobility of Te is particularly beneficial for improving the efficiency of the 2D PN photodiodes. We chose MoS_2_ as the N-type semiconductor to form the cathode terminal. The Te-MoS_2_ van der Waals heterojunction devices were fabricated by transferring mechanically exfoliated MoS_2_ few-layer flakes onto the as-synthesized Te flake. Figure 2f shows a schematic illustration of the Te-MoS_2_ van der Waals heterojunction device. Gold was used as the contacts to both terminals of the Te-MoS_2_ diode, and the heavily doped silicon substrate was used as the back-gate terminal. Figure 2g shows one representative SEM image of the heterojunction device after fabrication. The *I*–*V* characteristics of the Te-MoS_2_ diode in the dark were first measured by using a semiconductor parameter analyzer. By sweeping the bias applied between the anode (Te) and cathode (MoS_2_) from −2 to +2 V, the channel current *I*_D_ exhibits strong rectification behaviors (Figure 2h). The rectification ratio is highly tunable by changing the gate bias. Figure 2i shows the calculated rectification ratios obtained from the *I–V* curves at *V*_D_ = 2 V at different gate biases. With the gate voltages varying from −30 to +20 V, the rectification ratio increases from 128 to 3644. The current rectification can be explained by the energy band diagram in Figure 3a,b. Electrons in n-type MoS_2_ show a higher energy barrier at the Te/MoS_2_ interface under reverse bias. Under forward bias, this energy barrier is reduced and the current is mainly from the thermionic emission of electrons from MoS_2_ to Te. The diffusion of these electrons inside Te gives rise to the forward current.^1^ The rectification ratio exhibits a strong gate bias dependence. The forward current is more sensitively dependent on the gate bias. This is because, under forward bias, the thermionic emission of electrons depends on the electron density inside MoS_2_ near the interface (Figure 3c,d). By increasing the gate bias, the doping level of MoS_2_ and thus the electron density can be enhanced.

**Figure 4:**
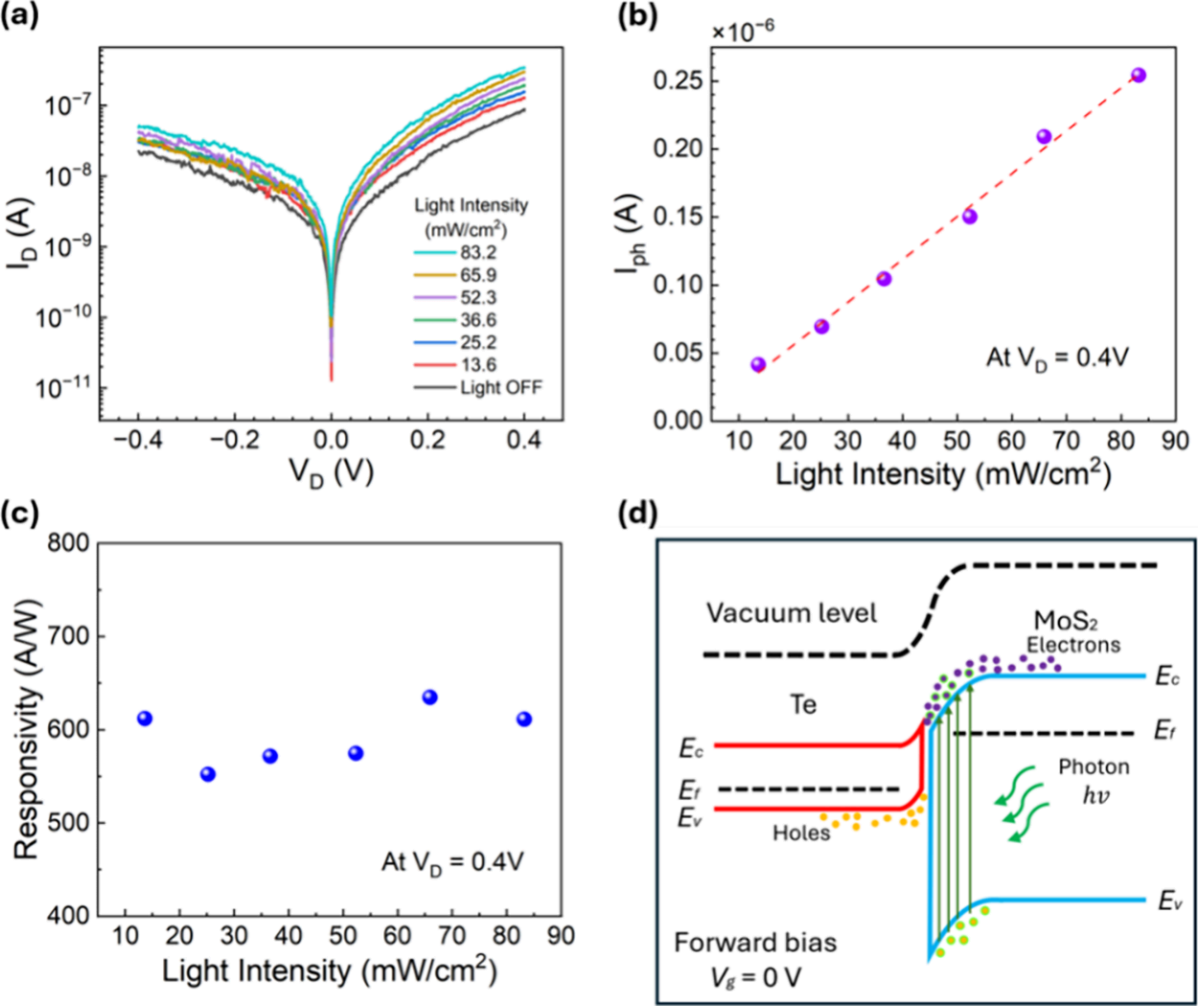
(a) Measured channel current *I*_D_ (on a logarithmic scale) as a function of channel bias of the Te-MoS_2_ heterojunction device under different light intensities. The gate bias is zero for all curves. (b) Photocurrent as a function of light intensity at channel bias of *V*_D_ = 0.4 V. (c) Calculated current responsivity as a function of light intensity at a channel bias *V*_D_ = 0.4 V. (d) Energy band diagram of the Te-MoS_2_ heterojunction photodiode.

The ultrathin nature of the Te-MoS_2_ heterostructure allows gate tunability of its diode *I*–*V* characteristics. It is a unique feature compared to conventional silicon-based PN diodes, for which the electrical properties are essentially fixed and determined by their predefined doping profiles. In order to investigate the gate tunability in photodetectors, we measured the photoresponses of the Te-MoS_2_ diode under different gate biases. A white-light optical beam illuminated the Te-MoS_2_ device in normal incidence. The detailed spectrum of the incident light was measured and is shown in the Supporting Information (Figure S1). We first measured the channel current *I*_D_ at zero gate bias by sweeping the channel bias *V*_D_ from −0.4 to 0.4 V and keeping the *V*_g_ to be zero, under incident light intensity ranging from 0 to 83.2 mW/cm^2^ (Figure 4a). Then, the photon current *I*_ph_ can be obtained by extracting the dark current. The current responsivity can be calculated as follows:
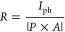
2where *I*_ph_ is the photocurrent, *P* is the incident light intensity, and *A* is the active area of the Te/MoS_2_ heterojunction (∼0.5 μm^2^). As shown in Figure 4b, the photocurrent linearly increases as the incident light power increases. We note that the forward biasing regime gives a higher responsivity than the reverse regime. Therefore, the current responosivity in Figure 4 was measured under a forward bias. The current responsivity under zero gate voltage is essentially independent of the incident light power in the range from 13.6 to 83.2 mW/cm^2^ (Figure 4c) when the gate bias is kept at zero_._ The average responsivity is about 593 A/W in this range of light power. This can be explained by the energy band diagram shown in Figure 4d. MoS_2_ is the photosensitive layer due to its band gap matching with the incident light frequency. The incoming photons generate electron hole pairs in the MoS_2_, and the electric field inside the depletion region is in favor of hole drifting from the interface to the MoS_2_ side. It contributes to a photocurrent that is in the same direction as the dark current of Te/MoS_2_ under a forward bias (Figure 3b). Such a unique Te-MoS_2_ band structure makes it different from the conventional silicon PN junction-based photodetectors, in which the photocurrent is in the opposite direction of dark current and its maximum responsivity occurs under reverse bias. The atomic thickness of MoS_2_ also minimizes the photocarrier recombination rate, which benefits the enhancement of the photocurrent and responsivity.

**Figure 5:**
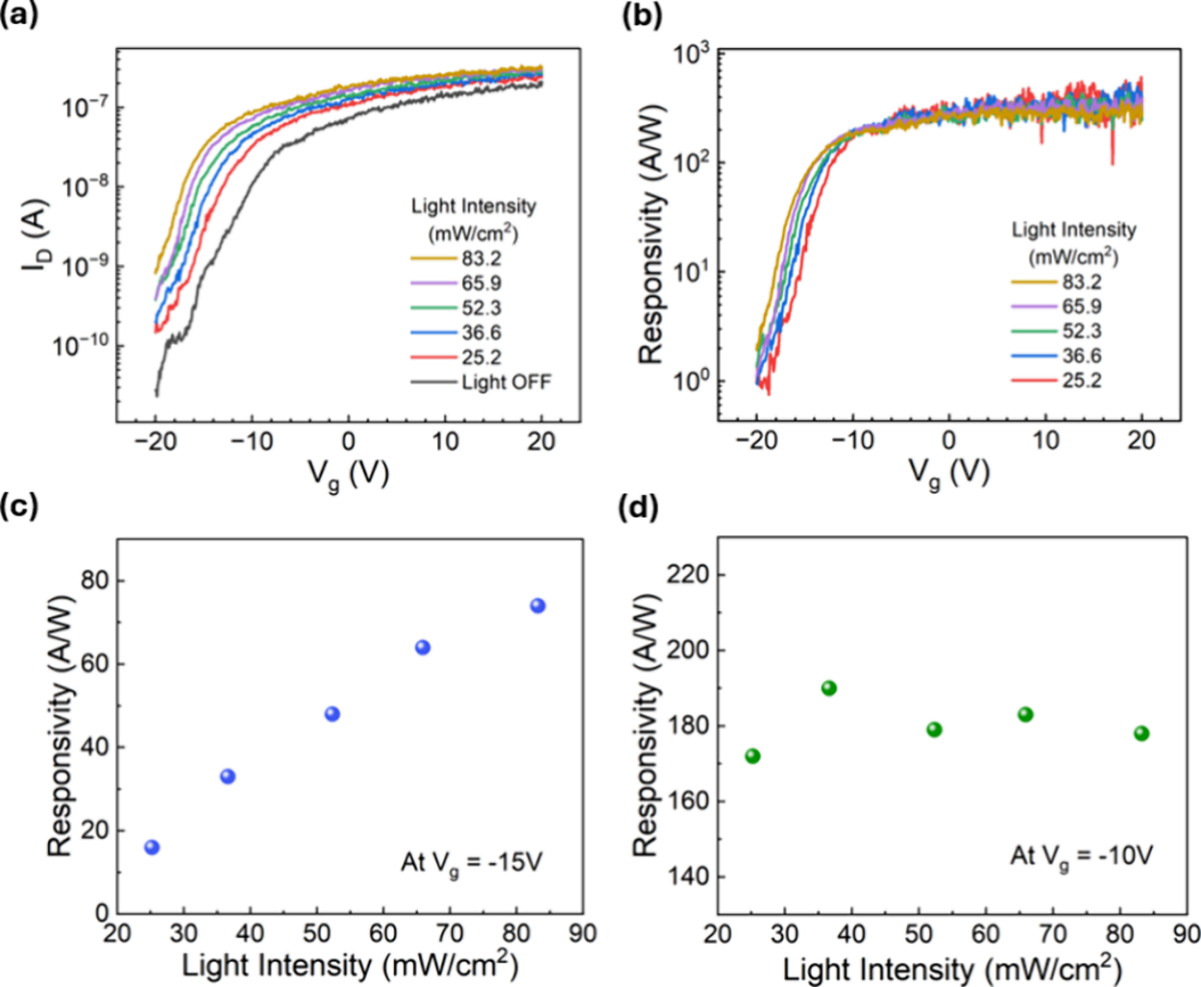
(a) Measured channel current *I*_D_ as a function of gate voltage *V*_g_ under various light intensities. The Te-MoS_2_ diode was biased under a forward bias *V*_D_ of 0.4 V. (b) Calculated responsivity as a function of gate voltage *V*_g_ obtained from the *I*–*V* curves in (a). Responsivity as a function of light intensity under gate voltages of −15 V (c) and −10 V (d).

Next, the photocurrent of the Te-MoS_2_ diode was measured at different gate biases in order to investigate its gate tunability. Figure 5a shows the *I–V* curves of the Te-MoS_2_ photodiode under 0.4 V constant forward bias and different incident light powers while sweeping the gate voltage *V*_g_ from −20 to +20 V. From the photocurrent measurement, we found that the current responsivity *R* of the Te-MoS_2_ photodiode increases as the gate bias increases for all tested light intensities (Figure 5b). It worth noting that the dependence of responsivity *R* on incident light intensity is different under different gate biases. At *V*_g_ = −15 V, the responsivity *R* increases linearly as the light intensity increases (Figure 5c). However, at *V*_g_ = −10 V and above, the responsivity exhibits a minimum dependence on the light intensity (Figure 5d). It indicates the existence of traps states in the Te/MoS_2_ heterostructure.^50,51^ When *V*_g_ = −15 V, most of the these trap states are empty, and a significant fraction of photogenerated carriers under low illumination is trapped and cannot contribute to the photocurrent. Only under higher incident light intensity, more trapped states are occupied and a higher photocurrent can be observed. At *V*_g_ = −10 V, the electron density in MoS_2_ increases, and a majority of the trap states are occupied. Consequently, the impact of trap states on the responsivity becomes much less significant. This is also consistent with the measurement results of Figure 4c.

## Conclusions

In conclusion, we presented a new PVD synthesis approach based on substrate engineering, which produces Te with a record-high field-effect hole mobility of 1450 cm^2^/(V s) among state-of-the-art synthesized 2D p-type semiconductors. We also demonstrated the Te-MoS_2_ PN heterojunction using the PVD-synthesized high-mobility Te. In the visible range, the Te-MoS_2_ exhibits photodetection capability with a current responsivity up to 630 A/W, which is about 1 order of magnitude higher than the one achieved using p-type Si-MoS_2_ PN photodiodes. The photoresponse of the Te-MoS_2_ photodiode also shows strong gate tunability and distinct optical characteristics that are different from conventional silicon-based photodetectors. The demonstrated Te-MoS_2_ photodetectors create new possibilities for the development of ultralight and highly tunable optoelectronic devices.

## Experimental Methods

### PVD Synthesis of 2D Tellurium Nanoflakes

Large hBN flakes (purchased from 2D Semiconductors) were mechanically exfoliated on a 2 × 0.5 in. silicon substrate initially. During the PVD process, a piece of the bulk Te precursor (from Sigma-Aldrich) was placed in a quartz boat located at the center of the PVD quartz tube as the evaporation source. The silicon substrates deposited with hBN flakes were then placed around 9 in. from the Te source downstream (Figure 1a). After the loading process, 900 sccm Ar and 100 sccm H_2_ were first flowed for about 4 min to purge the PVD tube. Then, a Lindberg/Blue M furnace (from Thermo Scientific) was used to heat up the tube to 660 °C from room temperature (22 °C) with a 40 °C/min ramp rate. A flow of 35 sccm Ar was introduced from one end of the tube during this heating process, and the Ar gas carried the vaporized Te onto the substrate for synthesis. The Te vapor condensed on the surface of the substrate with a lower temperature and formed nanoflakes. After reaching 660 °C, the flow of Ar gas was stopped, and the PVD tube was cooled immediately. The thickness of Te nanoflakes grown by this PVD process ranges from tens of nanometers to hundreds of nanometers.

### Device Fabrication

For the fabrication of Te transistors, the source and drain electrodes were defined by an electron beam lithography system (Elionix ELS-G100). Then, a layer of 50 nm gold (Au) was deposited by Lesker PVD-75 electron beam evaporation (EBE) at 1 Å/s followed by a lift-off process. For the Te-MoS_2_ heterojunction devices, the MoS_2_ (from 2D semiconductors) flakes were mechanically exfoliated onto poly(methyl methacrylate)/polyvinylpyrrolidone (PMMA/PVP), which were spin-coated on a silicon chip in advance. Next, the water-soluble PVP layer was dissolved by DI water, rendering the PMMA layer with MoS_2_ flakes separated from the silicon chip and floating on the DI water. This PMMA thin film was then transferred onto the Te flakes under a microscope in an aligned way. The PMMA film was subsequently dissolved by acetone. Contact electrodes were then made using the same process as used for the fabrication of Te transistors.

## Supporting Information Available

Detailed field-effect hole mobility calculation, photoresponse measurement settings, and spectrum of the light source (PDF)

## Abbreviations

BP: black phosphorus
CVD: chemical vapor deposition
PVD: physical vapor deposition
hBN: hexagonal boron nitride
FETs: field-effect transistors
AFM: atomic force microscopy
SEM: scanning electron microscopy
EBE: electron beam evaporation
PMMA: poly(methyl methacrylate)
PVP: polyvinylpyrrolidone
