# Supplementary material for: Physical Vapor Deposition of High-Mobility P-Type Tellurium and Its Applications for Gate-Tunable van der Waals PN Photodiodes
Source: ACS Appl Mater Interfaces. 2024 Dec 20;17(1):1861–8. doi: 10.1021/acsami.4c14865 (PMC11783348; doi:10.1021/acsami.4c14865)
Supplement: Supplementary file 1 — am4c14865_si_001.pdf [file am4c14865_si_001.pdf]

Supporting Information

Physical Vapor Deposition of High Mobility P-type Tellurium and its Applications for Gate-tunable van der Waals PN Photodiodes

Tianyi Huang[1], Sen Lin[2], Jingyi Zou[2], Zexiao Wang[1], Yibai Zhong[1], Jingwei Li[2], Ruixuan Wang[2], Zhixing Wang[2], Kevin St. Luce[2], Rex Kim[2], Jianzhou Cui[2], Han Wang[3], Qing Li[2], Min Xu[2, 4], Sheng Shen[1,2,5], Xu Zhang[1,2*]*

[1] Department of Mechanical Engineering, Carnegie Mellon University, Pittsburgh, PA, 15213, USA

[2] Department of Electrical and Computer Engineering, Carnegie Mellon University, Pittsburgh, PA, 15213, USA

[3] Ming Hsieh Department of Electrical and Computer Engineering, University of Southern California, Los Angeles, CA, 90089, USA

[4] Ray and Stephanie Lane Computational Biology Department, School of Computer Science, Carnegie Mellon University, Pittsburgh, PA, 15213, USA

[5] Department of Materials Science and Engineering, Carnegie Mellon University, Pittsburgh, PA, 15213, USA

Corresponding Author

Xu Zhang - Department of Electrical and Computer Engineering, Carnegie Mellon University, Pittsburgh, PA, 15213, USA; Email: xuzh@cmu.edu

Sheng Shen - Department of Mechanical Engineering, Carnegie Mellon University, Pittsburgh, PA, 15213, USA; Email: shengshe@andrew.cmu.edu

Mobility measurement and calculation:

The field effect mobility was extracted from the IV measurement shown in Figure 2d with a 0.5 V source drain voltage applied under room temperature. Following equation (1) $\mu = [dI_{ds}/dV_g] \times [L/WC_{gate}V_{ds}]$ The mobility was then calculated to be 1450 $cm^2V^{-1}s^{-1}$. C_{gate} was determined by equation $C_{gate} = [t_{SiO2}/(\varepsilon_0 \times \varepsilon_{SiO2}) + t_{h\text{-}BN}/(\varepsilon_0 \times \varepsilon_{h\text{-}BN})]^{-1} = 1.065\times10^{-4}$ Fm^{-2} where $\varepsilon_0 = 8.85\times10^{-12} Fm^{-1}$, $\varepsilon_{SiO_2} = 3.9$, $\varepsilon_{h\text{-}BN} = 3.5$ and SiO_2/hBN dielectric layer thickness $t_{SiO_2} = 285$ nm, $t_{h\text{-}BN} = 35$ nm.

Photoresponse measurement:

During the photoresponse measurement, a white light spot was vertically illuminated on the Te-MoS_2 heterojunction device. The diameter of the light spots is 0.35 cm, and the illumination area was then determined to be 0.096 cm^2. The light intensity was calculated by P = light power / illumination area, where the light power was measured by Thorlabs PM100D power meter. Figure S1 shows the spectrum of this white light source.

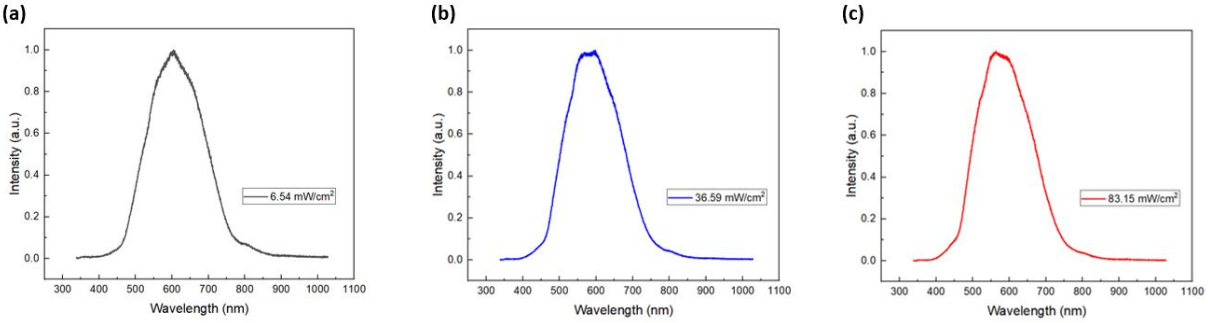

Figure S1. Spectrum of the light source under (a) 6.45 mW/cm^2, (b) 36.59 mW/cm^2 and (c) 83.15 mW/cm^2
